# Lutein and Zeaxanthin Are Positively Associated with Visual–Spatial Functioning in Older Adults: An fMRI Study

**DOI:** 10.3390/nu10040458

**Published:** 2018-04-07

**Authors:** Catherine M. Mewborn, Cutter A. Lindbergh, Talia L. Robinson, Marissa A. Gogniat, Douglas P. Terry, Kharine R. Jean, Billy Randy Hammond, Lisa M. Renzi-Hammond, Lloyd Stephen Miller

**Affiliations:** 1 Department of Psychology, The University of Georgia, Athens, GA 30602, USA; cmewborn@uga.edu (C.M.M.); cal@uga.edu (C.A.L.); talia.robinson25@uga.edu (T.L.R.); marissa.gogniat25@uga.edu (M.A.G.); kjean@uga.edu (K.R.J.); bhammond@uga.edu (B.R.H.); lrenzi@uga.edu (L.M.R.-H.); 2 Department of Physical Medicine and Rehabilitation, Department of Psychiatry, Massachusetts General Hospital, Boston, MA 02114, USA; douglasterry1@gmail.com; 3 Institute of Gerontology, Department of Health Promotions and Behavior, College of Public Health, The University of Georgia, Athens, GA 30602, USA

**Keywords:** xanthophylls, visual-spatial reasoning, fMRI, older adults, cognition

## Abstract

Lutein (L) and zeaxanthin (Z) are two xanthophyll carotenoids that have antioxidant and anti-inflammatory properties. Previous work has demonstrated their importance for eye health and preventing diseases such as age-related macular degeneration. An emerging literature base has also demonstrated the importance of L and Z in cognition, neural structure, and neural efficiency. The present study aimed to better understand the mechanisms by which L and Z relate to cognition, in particular, visual–spatial processing and decision-making in older adults. We hypothesized that markers of higher levels of L and Z would be associated with better neural efficiency during a visual–spatial processing task. L and Z were assessed via standard measurement of blood serum and retinal concentrations. Visual–spatial processing and decision-making were assessed via a judgment of line orientation task (JLO) completed during a functional magnetic resonance imaging (fMRI) scan. The results demonstrated that individuals with higher concentrations of L and Z showed a decreased blood-oxygen-level dependent (BOLD) signal during task performance (i.e., “neural efficiency”) in key areas associated with visual–spatial perception, processing, decision-making, and motor coordination, including the lateral occipital cortex, occipital pole, superior and middle temporal gyri, superior parietal lobule, superior and middle frontal gyri, and pre- and post-central gyri. To our knowledge, this is the first investigation of the relationship of L and Z to visual–spatial processing at a neural level using in vivo methodology. Our findings suggest that L and Z may impact brain health and cognition in older adults by enhancing neurobiological efficiency in a variety of regions that support visual perception and decision-making.

## 1. Introduction

Previous research has increasingly demonstrated the importance of diet and nutritional factors in brain health, especially in older adults [1]. Lutein (L) and zeaxanthin (Z) are two xanthophyll carotenoids that are acquired predominantly through the consumption of green leafy vegetables and brightly colored fruit and have previously been shown to positively relate to neurocognitive functioning [2]. Relative to other carotenoids present in human sera, L and Z preferentially cross the blood–brain barrier and accumulate in brain tissue, where they account for 66–77% of the total carotenoid concentration [3]. L and Z have been found to accumulate throughout diffuse regions of the brain, including the frontal, temporal, and occipital cortices, as well as the cerebellum and pons [3,4,5].

Historically, L and Z have been studied in relation to eye health, revealing their protective roles against age-related macular degeneration and the development of other optical diseases [6,7]. Previous literature has also demonstrated a positive relationship between intake of L and Z and better performance on a range of cognitive tasks, including executive functioning, learning and memory, verbal fluency, and processing speed [8,9,10,11,12]. Of particular relevance to this study, L and Z have been shown to positively relate to many aspects of visual–spatial functioning, including perceptual abilities, perceptual speed, and visual–spatial constructional skills [9,11]. Although this body of literature is still developing, there is evidence to suggest that L and Z may be two dietary factors that are important for a range of cognitive outcomes.

L and Z are primarily measured via concentrations in serum and the retina. Serum levels of L and Z are strongly related to recent dietary intake of foods rich in these nutrients [13]. In the retina, L and Z preferentially accumulate in the macula, where their concentrations can be assessed by measuring macular pigment optical density (MPOD), a validated measure of L and Z concentrations [14]. Because of the close relationship between the retina and the rest of the central nervous system, MPOD is considered a proxy for neural L and Z concentrations [5]. Although serum and retinal concentrations of L and Z are positively correlated, they are dissociable measures that may represent distinct aspects of dietary health [13,15,16]. For example, serum concentrations of L and Z are more variable than MPOD concentrations [17]. Additionally, after supplementation of L and Z, serum concentrations more quickly return to baseline levels whereas MPOD changes can last for several months [13]. Thus, serum concentrations of L and Z may more closely reflect acute dietary factors, whereas MPOD may reflect more stable L and Z levels that are acquired over a longer period of time.

Previous studies have demonstrated diffuse accumulation of L and Z in human brain tissue postmortem and have shown a positive relationship between concentrations of L and Z and performance on a wide range of cognitive tasks [2,3,4,8,9,10,11,12,18]. However, there is a lack of research investigating underlying neural mechanisms in vivo. To date, limited data exist that have examined the relationship between L and Z and neural structure and function using neuroimaging methodology. Our laboratory has conducted several investigations to address this gap in the literature using a sample of older adults enrolled in a nutrition intervention study. First, Lindbergh and colleagues (2017) assessed the cross-sectional relationship between serum and retinal concentrations of L and Z and brain activation during a verbal learning and memory fMRI task in our older adult sample [19]. The results indicated that greater L and Z levels were correlated with neural efficiency, as indicated by a decreased BOLD signal during task performance in several key brain regions for verbal learning and memory, including the central and parietal operculum, inferior frontal gyrus, supramarginal gyrus, planum polare, frontal and middle temporal gyrus, superior parietal lobule, pre- and post-central gyri, bilateral occipital cortex, and the cerebellum [19]. Another analysis from our laboratory, which used diffusion tensor imaging (DTI) in older adults, demonstrated that L and Z concentrations were positively correlated with measures of pre-intervention white matter integrity in major white matter tracts vulnerable to age-related decline, including the uncinate fasciculus and the cingulum [20]. Finally, in a pre-post intervention analysis of our sample, Lindbergh and colleagues (2018) found that L and Z supplementation increased cerebral perfusion and intensified neural responses in common brain regions at risk of deterioration in older adults, suggesting a possible neuroprotective effect of an L and Z intervention [21]. Other studies have also found L and Z to be related with better neural structure and function using structural MRI and EEG imaging technology [22,23]. Together, these findings offer a potential explanation for the mechanisms by which L and Z influence cognition, namely by bolstering neural structure and enhancing neural efficiency during cognitive task performance.

The current study aimed to further explore the mechanisms by which L and Z are related to cognitive functioning using functional magnetic resonance imaging (fMRI) in older adults. Given the importance of L and Z for visual health, we focused on the relationship between L and Z and performance during visual–spatial reasoning. 

Visual–spatial reasoning is a domain of cognitive function that involves an individual’s ability to perceive and organize visual stimuli. It includes a variety of related abilities, such as mental rotation, visual construction, and spatial orientation. Declines in visual–spatial abilities are associated both with normal [24,25] and pathological cognitive aging [26,27]. Visual–spatial reasoning is often impaired in the early stages of neurological disorders like Parkinson’s disease [28,29] and may be an indicator of disease progression in Alzheimer’s disease [30,31]. Additionally, visual–spatial abilities are important for functional competence in older adulthood [25,32]. For example, declines in spatial reasoning and orientation, in addition to memory and attention deficits, are associated with greater fall risk in older adults [33].

The Benton Judgment of Line Orientation (JLO) test is a commonly used measure of visual–spatial abilities that focuses specifically on spatial orientation, spatial judgment, and spatial reasoning [34]. Performance in visual–spatial reasoning tasks like the JLO is largely attributed to activation of right hemispheric structures [35,36,37,38]. Further exploration of neuroanatomical correlates of similar spatial reasoning tasks have consistently demonstrated evidence for the involvement of the right hemisphere, specifically the right parietal, parietal–temporal, and parietal–occipital regions [34,39]. More recent studies employing fMRI have consistently identified the right parietal and occipitoparietal involvement in JLO performance and similar orientation tasks, specifically activation in the right superior parietal cortex, angular gyrus, posterior supramarginal gyrus, and middle occipital gyrus [40,41,42]. Whereas parietal and occipital regions have been most consistently associated with performance in spatial orientation tasks, other regions have also been found to be associated with performance in visual–spatial reasoning tasks based on task difficulty. For example, Kesler and colleagues (2004) found that activation increased in frontal and occipital regions, in addition to the parietal region, as task difficulty in the JLO increased [41].

The right hemisphere is most closely associated with performance in visual–spatial tasks; however, left hemispheric involvement is also seen, particularly in older adults [41,43]. Previous work suggests that older adults compensate for age-related neural declines by recruiting more diverse brain regions and showing increased bilateralized brain activity while performing cognitively demanding tasks compared to the more focal activation seen in younger adults [43,44,45]. Although there has been limited work analyzing compensatory recruitment specifically during visual–spatial orientation tasks, there is evidence for differential activation and diminished specificity in regions of the brain related to processes such as visual recognition in healthy older adults compared to healthy young adults [46]. Other studies have similarly found diminished neural specificity in visual recognition and visual memory areas of the brain with increasing age [47,48]. 

In the current study, fMRI methodology was used to cross-sectionally assess the relationship between serum and retinal (MPOD) concentrations of L and Z and brain activity during performance on an fMRI-adapted JLO task [34] in a sample of community-dwelling older adults. In line with theories of compensatory brain aging, we hypothesized that L and Z concentrations would be negatively related to neural activity in parietal–temporal–occipital areas, including the superior parietal cortex, angular gyrus, supramarginal gyrus, and middle occipital gyrus, which have shown consistent involvement in performance of visual–spatial tasks [40,41,42]. In other words, individuals with lower concentrations of L and Z were expected to show a greater task-related BOLD signal (i.e., “neural inefficiency”) during JLO performance relative to individuals with higher concentrations. Generally, we hypothesized that activation would be right-lateralized, but could not rule out left hemispheric involvement [41,43]. Behaviorally, we hypothesized a positive relationship between L and Z concentrations and JLO task accuracy. Given that serum and retinal concentrations of L and Z are related, yet distinct, measures, we expected congruent, but not necessarily identical, relationships of these two measures with neural activation in the hypothesized regions. 

## 2. Materials and Methods

### 2.1. Subjects

The sample included 51 community-dwelling older adults (see Table 1 for demographic details). Participants were recruited as part of a larger, randomized-controlled trial of L and Z supplementation via newspaper advertisements, flyers, and a database of individuals who previously had consented to being contacted for future research studies. Data for this study are drawn from the baseline visits and are pre-supplementation. Exclusion criteria included left-handedness, history of ocular disease, corrected visual acuity of worse than 20:40 (Snellen notation), age-related macular degeneration in either eye, gastric conditions with the potential to interfere with L and Z absorption (e.g., gastric ulcer, Crohn’s disease, ulcerative colitis), contraindications for safe MRI data collection (e.g., metallic implants), or history of traumatic brain injury, dementia, or other neurological disease.

Data for this study were collected across three baseline visits, with an additional blood draw to collect serum data that was scheduled at a time convenient for participants. Prior to enrollment in the study, exclusion criteria were assessed via a telephone screening. Potentially eligible participants completed a physical examination to confirm good health and eligibility for continued participation. During visit one, full informed consent was obtained, demographic information and other variables pertinent to the larger study were collected, and cognitive status was screened for using the Clinical Dementia Rating Scale (CDR) [49]. Individuals were excluded from further participation if they showed evidence of mild to severe dementia, as indicated by a CDR total score of 1–3. During visit two, participants completed vision testing to collect MPOD measurements and other variables pertinent to the larger study. During visit three, participants completed MRI data collection.

Serum L and Z data were not available for 5 participants; thus, analyses using serum were conducted with a smaller sub-group of participants (*n* = 46). This sub-group did not differ significantly from the larger sample with regard to age, sex, education, mean MPOD, or mean performance on the JLO task.

### 2.2. Ethics

Informed consent was obtained from all participants. The study protocol was approved by the Institutional Review Board and the tenets of the Declaration of Helsinki were adhered to at all times by study personnel.

### 2.3. Methods

#### 2.3.1. Visual Acuity

Self-reported visual acuity was confirmed via Snellen acuity testing as delineated in Levenson and Kozarsky (1990) [50].

#### 2.3.2. Judgment of Line Orientation (JLO) Task

**Figure 1:**
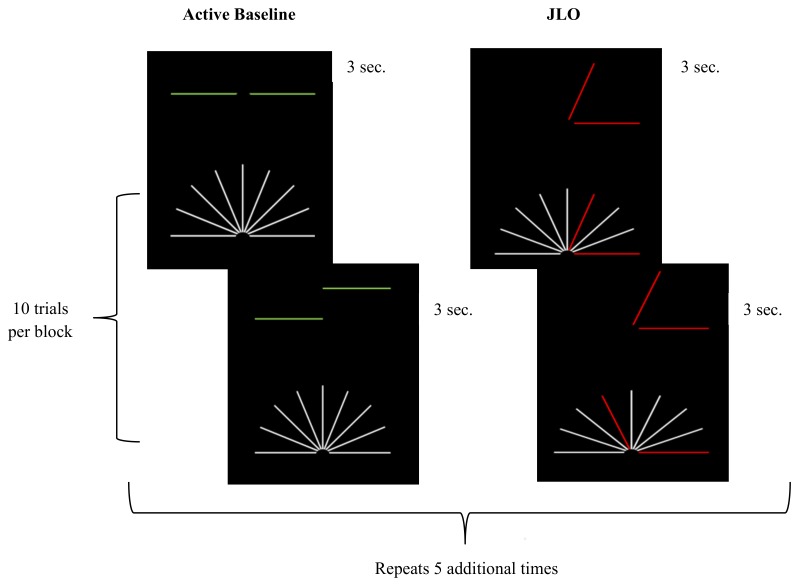
JLO task design. The figure provides a visual schematic of the judgement of line orientation (JLO) task. The two blocks (i.e., active baseline and JLO) were presented a total of six times, resulting in a total acquisition time of 6 min, 33 s. Ten stimuli were presented in each block in a random order with replacement for the active baseline and a sequential order from a library of 144 images for the JLO task.

Participants completed a JLO task conceptually based on the Benton JLO task [34] and adapted for the fMRI environment by the experimenters. The task was programmed using E-Prime (version 1.2, Psychology Software Tools, Inc., Pittsburgh, PA, USA) and presented through MRI compatible goggles (Resonance Technology Inc., Northridge, CA, USA). Prior to beginning the MRI, all participants were given a visual acuity test using the goggles to determine whether they could see the task clearly and accurately. Corrective lenses ranging from −9.0 to +3.0 were available to assist participants with myopia or hyperopia in viewing the task through the MRI compatible goggles. Participants responded using Cedrus Lumina LU400 MRI compatible response pads (Cedrus, San Pedro, CA, USA). The task consisted of 10 blocks each of active baseline and JLO task blocks (see Figure 1). During the active baseline blocks, participants were asked to assess whether two horizontal lines were in the same horizontal plane. Participants were instructed to respond using their right index finger if the two lines were horizontally even, or their left index finger if the two lines were uneven (maximum score = 30). Even and uneven line stimuli were presented in a random order with replacement. During the JLO task blocks, participants were asked to judge whether an angle presented in the top half of the screen matched an angle that was highlighted in red from an array of angles below. Participants were instructed to respond using their right index finger if the two angles matched, or their left index finger if the two angles did not match (maximum score = 30). During the JLO task blocks, stimuli were presented in sequential order from a library of 144 images. During each active baseline and JLO task block, ten stimuli were presented for three seconds each. Participants were trained on the JLO task outside of the scanner for approximately 30 min to ensure understanding of the task and task response instructions.

#### 2.3.3. Macular Pigment Optical Density (MPOD)

MPOD was assessed via customized heterochromatic flicker photometry (cHFP) using well-validated procedures [14,51,52]. Participants were shown a disc that was comprised of shortwave “blue light” (460 nanometer, nm) and midwave “green” light (570 nm). The two wavelengths of light are presented in square-wave, counter-phase orientation, which causes the stimulus disc to appear to “flicker.” Participants adjust the intensity of the 460 nm light until it matches the luminance of the 570 nm light and the disc ceases to “flicker.” Since the macular pigment strongly absorbs the 460 nm light, individuals with a denser macular pigment layer (thus a higher concentration of retinal L and Z) require more intense 460 nm to match the 570 nm light. This procedure was conducted in both the foveal and parafoveal regions of the retina and was customized for each individual participant based on their previously measured critical flicker fusion frequency (CFF). MPOD was calculated as the log intensity of the 460 nm light required to match the 570 nm light in the fovea versus the parafovea regions.

#### 2.3.4. Serum Lutein and Zeaxanthin (Serum L & Z)

Full details of the serum L & Z analysis can be found elsewhere [19]. Briefly, 7 mL (mL) of blood was collected by a certified phlebotomist. Blood samples were placed on ice and centrifuged for 15 min after collection. Serum data were extracted using standard lipid extraction methods, and serum was frozen in 1 mL cryotubes at −80 °C until analysis. L and Z concentrations were analyzed using a Hewlett Packard/Agilent Technologies 1100 series high performance liquid chromatography (HPLC) system with a photodiode array detector (Agilent Technologies, Palo Alto, CA, USA). A 5 μm, 200 A° polymeric C_30_ reverse-phase column (Pronto-SIL, MAC-MOD Analytical Inc., Chadds Ford, PA, USA) was used to separate the analytes. Initially, serum L and Z were quantified separately. Serum L levels (μmol/L) were added to serum Z levels (μmol/L) to create a combined serum L & Z value that was used in all analyses. 

#### 2.3.5. Neuroimaging Acquisition

MRI data were acquired using a General Electric Signa HDx 3T MRI scanner (GE; Waukesha, WI, USA). Images were acquired axially and covered from the top of the head to the brainstem. All slices were aligned to the anterior–posterior commissure line. Three-dimensional (3D) structural scans were collected using a high-resolution, T1-weighted fast spoiled gradient recall echo sequence (TR = 7.5 ms, TE = < 5 ms; FOV = 256 × 256 mm matrix; flip angle = 20°; slice thickness = 1.2 mm; 154 slices). The acquisition of the 3D structural scan lasted 6 min, 20 s. fMRI scans were collected using a T2-weighted single shot echo planar imaging (EPI) sequence (TR = 1500 ms, TE = 25 ms; 90° RF pulse; acquisition matrix = 64 × 64; FOV = 220 × 220 mm; slice thickness = 4 mm; 30 interleaved slices). Acquisition of the fMRI scans lasted 6 min, 33 s. Additionally, a pair of magnitude and phase images were acquired for use in fieldmap-based unwarping of the fMRI scans (TR = 700 ms, TE = 5.0/7.2 ms; FOV = 220 × 200 mm matrix; flip angle = 30°; slice thickness = 2 mm; 60 interleaved slices). The acquisition of the magnitude and phase images lasted 2 min, 20 s. 

#### 2.3.6. Data Analysis

fMRI scans were processed using Statistical Parametric Mapping (SPM12) [53]. Data were converted from GE DICOM format to NIFTI format using the dcm2nii conversion tool [54]. Pre-processing followed a standard procedure, including slice time correction to adjust for interleaved acquisition and realignment of the functional images to correct for motion. Fieldmaps were calculated from the acquired magnitude and phase images and applied to functional images to correct for distortion. Three-dimensional (3D) structural scans were co-registered to the functional scans and images were transformed into Montreal Neurological Institute (MNI) standard space. Deformation fields were created and applied to the functional images for spatial normalization to MNI standard space. Three-dimensional (3D) structural scans were segmented to separate brain tissue from cerebrospinal fluid, bone, non-brain soft tissue, and air. Images were smoothed using a 6.75 mm FWHM Gaussian filter. 

Activation maps of JLO performance minus active baseline were created using the general linear model found in SPM12, with a statistical threshold of *p* < 0.001, family-wise error (FWE) corrected and a minimum of eight continuous voxels. These thresholds were chosen to balance between risk of Type I and Type II error rates [55]. To assess the relationship between MPOD and neural activity within the hypothesized regions, a simple regression analysis was conducted, with the activation maps as the dependent variables and MPOD as the predictor variable. Analyses were repeated with serum L & Z as the predictor variable. 

The relationship of L and Z concentrations with accuracy on the JLO task were evaluated in the Statistical Package for Social Sciences (IBM SPSS Version 21.0) using regression analyses, with measures of L or Z (MPOD or serum) as the independent variable and number of correct responses during the JLO condition as the dependent variable. Given that MPOD and serum L & Z were directionally hypothesized to positively relate to task accuracy, one-tailed statistical tests were conducted.

## 3. Results

### 3.1. JLO Behavioral Performance

Demographic and performance descriptive statistics can be found in Table 1. Due to a software error, performance data for the JLO task were lost for 11 participants. Of note, these participants did not significantly differ from the larger sample with regard to age, sex, education, mean MPOD, or mean serum L & Z concentration. MPOD (*n* = 40) did not significantly predict performance accuracy during the JLO condition, although results neared statistical significance (*R*^2^ = 0.07, F = 2.841, *p* = 0.05). Similarly, serum L & Z (*n* = 39) also did not significantly predict accuracy during the JLO condition (*R*^2^ = 0.05, F = 1.776, *p* = 0.10). 

### 3.2. Whole-Brain Analysis

**Figure 2:**
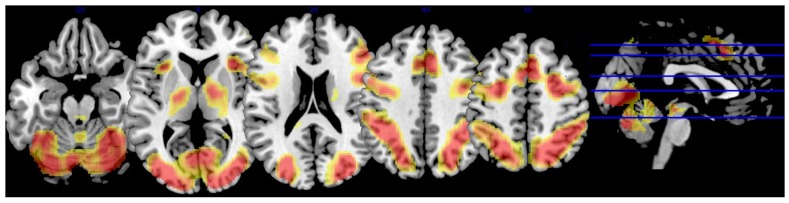
Whole brain activation during JLO task. The figure depicts brain activation during the JLO task minus active baseline contrast (independent of lutein and zeaxanthin levels). Activation superimposed on a single-subject anatomical template in MNI space provided by MRIcron [56]. To conserve space, only five slices were selected to showcase task-related BOLD response based on the largest extent activation.

The JLO task minus active baseline contrast (*p* < 0.001, FWE corrected, minimal voxel cluster = 8) revealed widespread activation in regions commonly associated with vision, motor coordination, and visual–spatial decision-making, including the bilateral superior and inferior lateral occipital cortexes, right occipital pole, right occipital fusiform gyrus, right superior parietal lobule, bilateral paracingulate gyrus, left cingulate gyrus, bilateral middle frontal gyrus, bilateral frontal pole, bilateral precentral gyrus, bilateral insular cortex, and cerebellar vermis (see Table 2 and Figure 2).

### 3.3. Lutein and Zeaxanthin Analysis

#### 3.3.1. MPOD

**Figure 3:**
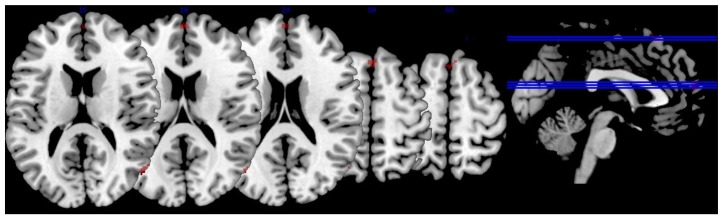
Relationship between lutein and zeaxanthin and brain activation. The figure depicts brain activation during the JLO task minus active baseline contrast that was significantly and negatively related to macular pigment optical density (MPOD) levels. In other words, individuals with lower levels of lutein and zeaxanthin showed an increased BOLD signal in these regions (i.e., “neural inefficiency). The activation is superimposed on a single-subject anatomical template in MNI space provided by MRIcron [56]. To conserve space, only five slices were selected to showcase task-related BOLD response based on the largest extent activation.

Following the JLO task, minus the active baseline contrast (*p* < 0.001, FWE corrected, minimal voxel cluster = 8), MPOD negatively predicted brain activity in regions associated with visual–spatial performance and decision-making, including the right superior and inferior lateral occipital cortexes, right angular gyrus, bilateral cingulate gyrus, right middle frontal gyrus, right frontal pole, right superior frontal gyrus, and right precentral gyrus (*p* < 0.01, see Table 3 and Figure 3). In other words, lower MPOD was associated with greater activation (i.e., “neural inefficiency”) in these regions. As indicated in Table 3, the effect sizes for the relationship between MPOD and brain activation during the JLO task ranged from r = 0.343 to r = 0.403.

#### 3.3.2. Serum L & Z

**Figure 4:**
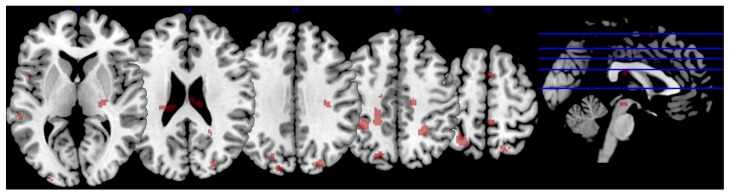
Relationship between lutein and zeaxanthin and brain activation. The figure depicts brain activation during the JLO task minus active baseline contrast that was significantly and negatively related to serum lutein and zeaxanthin levels. In other words, individuals with lower levels of lutein and zeaxanthin showed an increased BOLD signal in these regions (i.e., “neural inefficiency). The activation is superimposed on a single-subject anatomical template in MNI space provided by MRIcron [56]. To conserve space, only five slices were selected to showcase task-related BOLD response based on the largest extent activation.

Serum L & Z also negatively predicted brain activity in regions associated with visual–spatial performance, motor coordination, and decision-making after subtraction of the active baseline (*p* < 0.001, FWE corrected, minimal voxel cluster = 8), including the bilateral superior lateral occipital cortex, left occipital pole, bilateral middle temporal gyrus, bilateral superior temporal gyrus, left superior parietal lobule, left planum temporale and planum polare, bilateral temporal fusiform cortex, left temporal pole, right posterior supramarginal gyrus, right lingual gyrus, left central opercular cortex, left parahippocampal gyrus, right thalamus, bilateral precentral gyrus, right postcentral gyrus, and left superior frontal gyrus (*p* < 0.01, see Table 4 and Figure 4). Thus, lower serum concentrations of L & Z were associated with greater activation and increased neural inefficiency in these regions. As indicated in Table 4, effect sizes for the relationship between serum L & Z and brain activation during the JLO task ranged from r = 0.360 to r = 0.492.

## 4. Discussion

Although historically studied in relation to eye health [7], accumulating literature has demonstrated the potential for the xanthophyll, lutein (L), and its isomer, zeaxanthin (Z), to benefit a range of neurocognitive functions as well, particularly in older adults [2]. The present study is among the first attempts to investigate underlying neural mechanisms in vivo, using fMRI.

Initial whole brain analyses revealed a pattern of activation that is consistent with prior neuroimaging studies involving JLO and visual–perception more broadly, including occipito–temporal and parieto–frontal networks [40,41,42,57,58]. As hypothesized, both retinal (i.e., MPOD) and serum L and Z were negatively correlated with brain activity in several regions during JLO performance, though a somewhat different pattern of results was observed for the two measures. Although both MPOD and serum L and Z concentrations were associated with a BOLD signal in primary visual and spatial processing areas (e.g., lateral occipital cortex and occipital pole), a lower MPOD tended to more consistently relate to increased activation in frontal regions with demonstrated involvement in visual–perceptual performance, such as in the right superior frontal and right middle frontal gyri and the frontal pole [58,59]. Serum L and Z seemed to be more related to temporal–parietal regions associated with visual–spatial processing (e.g., the superior and middle temporal gyri, superior parietal lobule) in addition to primary visual areas. Thus, MPOD, which represents more stable L and Z concentrations acquired over time, may improve performance during visual–spatial tasks by increasing the efficiency of key frontal decision-making regions. In a complementary fashion, serum L and Z, which represent more acute dietary intake of nutrients, may improve performance by increasing the efficiency of visual–spatial processing. 

It is not uncommon for the aging brain to show increased frontal involvement during cognitive performance, and consistent with the posterior–anterior shift in aging (PASA) model, this may reflect a form of compensation for age-related deterioration in other brain regions (e.g., occipitotemporal) [60]. Importantly, the compensatory PASA pattern has been specifically observed during visual–spatial perception and processing [61,62]. Individuals whose long-term dietary patterns include greater consumption of L and Z, as reflected by MPOD, may be especially buffered by general age-related neuropathological processes and thus require less frontal compensation. This interpretation is consistent with research demonstrating that older adults who can maintain healthy, “younger” brains (e.g., greater gray matter density) require less recruitment of additional neural circuitry (e.g., prefrontal areas) to carry out cognitive tasks relative to less successfully aging peers [63]. The ability to adequately complete a cognitive task with more focused cerebral involvement has previously been interpreted as “neural efficiency” [64] and seems consistent with the pattern observed here, likely due to the prophylactic effects of the L and Z carotenoids in neural tissue [65].

Although the precise role of frontal areas in the context of visuospatial performance remains to be fully elucidated, it has long been speculated to encompass a range of functions, such as decision-making, online maintenance of visual information necessary to make judgments, and more general cognitive control [58,66,67]. Observations in previous studies where “difficult” JLO items elicit prefrontal cortex activation, whereas “easy” items (e.g., those containing fewer line foils) do not have been interpreted as support for the possibility that complex visual–perceptual judgments require aspects of executive function [41,42]. Long-term dietary patterns involving L and Z intake, measured via MPOD, may improve the efficiency of neural processes underlying these higher order executive functions. Our findings are consistent with, and may help to explain, previous studies demonstrating a positive relationship between visual system ability and executive skills in older adults [68].

Contrary to expectations, L and Z levels were not significantly related to JLO accuracy in behavioral measures, although MPOD neared significance as a predictor. However, the findings were in the expected direction: individuals with higher MPOD and serum L & Z concentrations displayed relatively more accurate performances on the JLO task. Although there was a range in performance, accuracy on the JLO task (scores = 23–59), the overall mean accuracy was high (M = 52.53/60, SD = 4.49) with most participants scoring better than 87%. The high overall average could have a limited association with L and Z concentrations due to a “ceiling effect.” Alternatively, or perhaps in combination, it is possible that an analysis with a larger sample and correspondingly greater statistical power would reveal a significant relationship. 

Another explanation for the lack of significant correlation between L and Z concentrations and behavioral JLO performance lies in theories of compensatory aging, which suggest that there may be neural changes in the way older adults process information (e.g., recruitment of additional neural areas, increased neural activation) that allow them to maintain a high level of cognitive performance, and that nutritional factors contribute to these compensatory processes [69,70]. Our results showed that lower L and Z concentrations are associated with greater neural activation or neural “inefficiency” despite no statistically significant correlations with behavioral JLO performance. While older adults with lower L and Z concentrations appear to have maintained a high level of cognitive performance, they require greater compensatory neural activity than individuals with high L and Z concentrations. Thus, L and Z may contribute to “youthful” and efficient brain functioning regardless of cognitive outcomes.

A notable limitation of the present study is its cross-sectional nature, which prevents conclusions regarding the directionality of the observed association between L and Z and neurocognitive performance. Although findings from previous longitudinal studies have suggested that consuming carotenoids reduces the risk of age-related neurodegenerative diseases across time [71], only a few randomized controlled trials have evaluated the effects of L and/or Z supplementation in older adults [21,72]. As noted in recent reviews [73], additional randomized controlled trials are needed to determine whether low L and Z consumption contributes to declines in brain health and cognition or perhaps represents a consequence, such as poor nutritional choices resulting from cognitive impairments. In addition, the present study was restricted to a purely Caucasian sample that tended to be well educated and cognitively healthy. Replication in a more diverse sample is warranted to evaluate the generalizability of the observed findings.

Despite these limitations, the present study represents an important contribution to the literature and marks the first attempt to investigate neural mechanisms underlying the relationship between L and Z and visual–spatial functioning using fMRI. Findings suggest that L and Z may promote brain health and cognition in older adults by enhancing neurobiological efficiency in a variety of regions that support visual perception and decision-making. More broadly, the present study bridges the gap between the disciplines of nutrition and neuroscience to advance our understanding of the critical relationship between diet and brain function. Dietary features such as L and Z appear to hold considerable potential as modifiable and inexpensive lifestyle factors to promote neurocognitive health in the rapidly expanding older adult segment of the population.

## Figures and Tables

**Table 1:** Descriptive characteristics of participants, task performance, and lutein/zeaxanthin concentrations.

Age (Years)	Sex (% Female)	Race (% Caucasian)	Education (Years)	JLO Task Accuracy (Max = 60) ^1^	MPOD (o.d.)	Serum L & Z (μmol/L) ^2^
71.75 ± 6.16	58.8	100.0	16.1 ± 3.15	52.53 ± 4.49	0.495 ± 0.177	0.321 ± 0.170

JLO = Judgement of Line Orientation. MPOD = macular pigment optical density. o.d. = optical density, the log ratio of transmitted light passing through the macula. ^1^ Data available for 40 participants. ^2^ Data available for 46 participants.

**Table 2:** Whole-brain activation during the JLO task.

Region	*x*	*y*	*z*	Extent	T-Score
L superior lateral occipital cortex	−32	−88	12	35,624	23.75
L inferior lateral occipital cortex	−34	−88	2	*	21.38
	−38	−86	0	*	21.21
	−42	−64	12	*	20.35
R superior lateral occipital cortex	34	−80	22	*	21.63
	22	−64	52	*	21.58
	38	−78	12	*	20.03
	26	−72	36	*	18.76
	28	−74	30	*	18.40
R inferior lateral occipital cortex	34	−88	0	*	19.59
	38	−84	4	*	19.18
	40	−84	−4	*	18.52
R occipital pole	18	−96	6	*	18.81
R superior parietal lobule	32	−52	46	*	18.38
L cerebellum, vermis VI	−4	−72	−26	*	18.31
R occipital fusiform gyrus	38	−70	−10	*	18.06
R paracingulate gyrus	6	20	44	13,072	18.85
L paracingulate gyrus	−6	14	46	*	14.77
	−8	24	38	*	12.37
R middle frontal gyrus	28	0	52	*	17.54
	46	24	24	*	14.25
	50	30	28	*	13.01
L middle frontal gyrus	−26	−2	52	*	13.90
	−38	28	22	*	9.60
R insular cortex	32	20	−2	*	17.48
L insular cortex	−34	20	0	*	15.28
R precentral gyrus	42	6	28	*	17.15
L precentral gyrus	−32	−4	48	*	14.83
	−46	4	30	*	13.30
R frontal pole	44	44	−14	*	13.23
	38	58	0	*	11.16
	46	50	−8	*	10.16
L cingulate gyrus	−6	0	26	75	8.24
L frontal pole	−46	48	−4	30	8.05
	−48	44	−8	*	7.63
	−42	52	6	*	7.06

The table above reports whole-brain activation during the JLO task minus active baseline contrast (*p* < 0.001, family-wise error (few) corrected, minimal voxel cluster = 8). R = right hemisphere. L = left hemisphere. *x*, *y*, and *z* coordinates are in Montreal Neurological Institute (MNI) space (mm). * = cluster overlap with preceding row.

**Table 3:** Relationship between MPOD and brain activation.

Region	*x*	*y*	*z*	Extent	T-Score	Effect Size (r)
R superior lateral occipital cortex	54	−70	16	19	3.08	0.403
	56	−66	16	*	2.90	0.383
R inferior lateral occipital cortex	56	−68	12	*	2.94	0.387
R middle frontal gyrus	48	18	30	59	3.03	0.397
R frontal pole	0	60	16	31	2.93	0.386
L cingulate gyrus	−2	4	24	30	2.85	0.377
R cingulate gyrus	4	2	30	*	2.56	0.343
R angular gyrus	60	−50	38	15	2.84	0.376
	62	−50	34	*	2.81	0.373
R precentral gyrus	62	12	8	9	2.82	0.374
R superior frontal gyrus	6	56	25	10	2.62	0.351

The table above reports brain activation that was significantly and negatively associated with MPOD during the JLO task minus the active baseline contrast (*p* < 0.01). MPOD = macular pigment optical density. R = right hemisphere. L = left hemisphere. *x*, *y*, and *z* coordinates are in MNI space (mm). * = cluster overlap with preceding row.

**Table 4:** Relationship between serum L & Z and brain activation.

Region	*x*	*y*	*z*	Extent	T-Score	Effect Size (r)
R precentral gyrus	16	−18	48	62	3.75	0.492
L middle temporal gyrus	−56	−8	−12	51	3.55	0.472
L superior parietal lobule	−36	−42	50	199	3.46	0.462
	−28	−56	58	*	3.29	0.444
L superior lateral occipital cortex	−20	−74	40	61	3.42	0.458
	−16	−88	36	12	2.73	0.381
R temporal fusiform cortex	36	−30	−16	33	3.36	0.452
L precentral gyrus	−20	−40	44	68	3.32	0.448
R superior lateral occipital cortex	26	−84	30	67	3.21	0.436
	24	−62	56	51	3.04	0.417
L superior frontal gyrus	−22	30	54	28	3.16	0.430
L posterior superior temporal gyrus	−60	−32	4	49	2.92	0.403
L planum temporale	−62	−22	6	*	2.70	0.377
L superior temporal gyrus	−52	−38	2	*	2.56	0.360
L occipital pole	−28	−96	6	9	2.99	0.411
L temporal fusiform cortex	44	−16	−16	9	2.95	0.406
R thalamus	24	−22	4	36	2.93	0.404
L planum polare	−36	−8	−10	19	2.87	0.397
R posterior supramarginal gyrus	46	−38	10	21	2.58	0.362
R posterior superior temporal gyrus	54	−36	8	*	2.87	0.397
L Heschl’s gyrus	−46	−22	2	12	2.83	0.392
L parahippocampal gyrus	−32	−36	−18	22	2.83	0.392
R postcentral gyrus	6	−40	62	22	2.66	0.372
L central opercular cortex	−48	6	2	11	2.75	0.383
R anterior middle temporal gyrus	58	−2	−22	19	2.70	0.377
R anterior superior temporal gyrus	50	−2	−16	*	2.62	0.367
L temporal pole	−52	6	−18	10	2.67	0.373
R lingual gyrus	18	−60	−16	8	2.61	0.366

The table above reports brain activation that was significantly and negatively associated with serum L & Z during the JLO task minus active baseline contrast (*p* < 0.01). L = lutein. Z = zeaxanthin. *x*, *y*, and *z* coordinates are in MNI space (mm). * = cluster overlap with preceding row.

## Registry Information

ClinicalTrials.gov number, NCT02023645.
